# The Church's Attitude Towards Disease

**Published:** 1917-06-23

**Authors:** 


### THE CHURCH'S ATTITUDE TOWARDS DISEASE

FROM A CORRESPONDENT.

Is it not time that the Church of England gave us a definite pronouncement on the subject of religion and disease? It is within living memory that the chief obstacle to sanitation was men's persuasion that cholera and typhus were "visitations" from the hand of God Himself, and that all interference with their rise or spread was impious. Kingsley's works are full of references to this spirit, which blocked every effort at reform in many towns and country-places sixty years ago. At the present day the battle is partly won. Yet even now the doctor is liable to meet with patients who resist every impulse towards returning health, and passively nullify all his treatment by the fixed idea that the complaint from which they suffer has been sent by the will of God, and to it they must patiently submit. It would seem that it is time for the Church to repudiate altogether the doctrine, unfortunately set forth in the Prayer-Book, that disease is of God. Do we, or do we not, believe that plague and grievous sickness are a punishment for sin, or are sent by Him to "try the patience" of the sick, with the purpose of correcting or amending "whatsoever doth offend in them"? Do we now believe, and ought we to believe, that illness is "sent"? Every discovery of medical science forbids the supposition. The most rigid investigation fails to show that the burden of disease falls more heavily on the wicked than on the good. On the contrary, by far the heaviest toll is taken by disease from the young and innocent. Science and morality alike are opposed to the teaching which has poisoned the wells of Christianity so long—a mediæval inheritance unsupported by the example of Christ. For it is very plain to be seen that Christ, who mirrored the mind of the Creator, regarded disease as an evil to be overcome by every force which could be enlisted against it. On no occasion did He refuse to heal, nor was He ever found to preach the virtue of being ill. It may indeed be said that He did not suffer disease in any form to subsist in His presence. It is very advisable to purge the Prayer-Book, as Convocation is now doing, of archaisms such as are no longer understood by the people of this generation; but it is far more vital to men's faith to clear the ground once for all from the bad reasoning and false analogies which have led men into the quagmire of praying to a God who "plagues us with divers diseases and sundry kinds of death." If the trumpet give an uncertain sound, who shall prepare himself to the battle?

